# Kanglaite (Coix Seed Extract) as Adjunctive Therapy in Cancer: Evidence Mapping Overview Based on Systematic Reviews With Meta-Analyses

**DOI:** 10.3389/fphar.2022.901875

**Published:** 2022-08-12

**Authors:** Cuncun Lu, Shuilin Wu, Lixin Ke, Fumei Liu, Wenru Shang, Xiuxiu Deng, Yanli Huang, Qiang Zhang, Xin Cui, Alexios-Fotios A. Mentis, Yanming Xie, Zhifei Wang

**Affiliations:** ^1^ Institute of Basic Research in Clinical Medicine, China Academy of Chinese Medical Sciences, Beijing, China; ^2^ Evidence-Based Social Science Center, School of Public Health, Lanzhou University, Lanzhou, China; ^3^ Hepatobiliary and Pancreatic Center, The First Affiliated Hospital, Sun Yat-sen University, Guangzhou, China; ^4^ Evidence-Based Medicine Center, School of Basic Medical Sciences, Lanzhou University, Lanzhou, China; ^5^ Department of Gastroenterology, Chengdu Pidu District Hospital of Traditional Chinese Medicine, Chengdu, China; ^6^ University Research Institute of Maternal and Child Health and Precision Medicine, National and Kapodistrian University of Athens, Athens, Greece

**Keywords:** kanglaite, cancer, meta-analyses, methodological quality, AMSTAR 2

## Abstract

**Background:** Several quantitative systematic reviews of Kanglaite (KLT), an herb preparation used to treat cancer and malignant pleural effusion, have been published in recent years. However, the clinical evidence reported in these studies has not been pursued further and the methodological quality of these meta-analyses remains unknown. Therefore, an overview was designed to map the evidence landscape based on the published meta-analyses on KLT in cancer treatment.

**Methods:** Two bibliographic databases (PubMed and Embase) were searched from inception to 25 November 2021. Two independent reviewers were involved in study selection, data abstraction, and methodological quality assessment using AMSTAR 2. The principal features of publications and the clinical outcomes of efficacy and safety were synthesized narratively, and results of methodological quality were reported as frequencies and percentages with the corresponding 95% confidence intervals. The evidence map was used to visualize the overall quality. Excel 2016 and Stata 17/SE were used for data analysis.

**Results:** Thirteen meta-analyses published in English were included for in-depth analysis. Among them, the year of publication ranged from 2008 to 2021, and the number of included patients ranged from 488 to 2,964. Regarding the cancer type, seven articles focused on non-small cell lung cancer, two on malignant pleural effusion, and four reviews on digestive system malignancies, such as hepatocellular carcinoma and pancreatic cancer. Almost all included meta-analyses reported that KLT as adjunctive therapy could improve various efficacy outcomes (such as disease response rates, quality of life, immune indicators) and reduce the rate of occurrence of adverse reactions, such as nausea and vomiting, leukopenia, and anemia. In terms of their methodological quality, three meta-analyses were of low quality, whereas 10 studies were critically low in quality. The methodological flaws main involved items 2 (“predesigned protocol and registration informatio’’), 3 (“rationale of study design for inclusion”), 4 (“comprehensive search strategy’’), 5 (“literature selection in duplicate’’), 7 (“list of excluded studies with reasons’’), 8 (“adequate information on included studies’’), 10 (“funding support for included primary studies’’), and 12 (“evaluation of the potential impact of risk of bias’’) based on the AMSTAR 2 tool.

**Conclusion:** Current evidence reveals that KLT is effective and safe as an adjunctive treatment for non-small cell lung cancer, malignant pleural effusion, and digestive system malignancies (such as hepatocellular carcinoma). However, the results assessed in this overview should be further verified using well-designed and clearly reported clinical trials and meta-analyses of KLT.

## Introduction

Cancer adversely influences the health and quality of life of affected individuals, and cancer deaths account for 17% of all deaths worldwide (Wei et al., 2022). Although chemotherapy is currently the primary intervention for most common cancers such as those of the lungs, stomach, and liver, serious adverse reactions and multidrug resistance limit the use and efficacy of chemotherapy in clinical practice (Lu et al., 2021a; Wei et al., 2022). Traditional Chinese medicine, usually regarded as a type of complementary and alternative medicine, shows promise in providing a supplementary therapeutic pathway for medical oncologists to assist patients with cancer (Wang et al., 2020a; Liu et al., 2020). According to a recent narrative review (Liu et al., 2020), traditional Chinese medicine for cancers mainly consists of six therapeutic principles, including 1) reinforcing health and eliminating pathogens, 2) clearing heat and removing toxins, 3) activating blood and resolving stasis, 4) softening hardness and dissipating mass, 5) resolving phlegm and removing dampness, and 6) nourishing the heart and tranquilizing the mind.

Kanglaite (KLT), a Chinese medicine preparation, is widely used in China to treat lung and liver cancer, or complications of cancers, such as malignant pleural effusion (Liu et al., 2019a; Huang et al., 2020; Zhu et al., 2021). This preparation is available as injections and capsules, and it is mainly composed of the oil extracted from Coix seeds (*Coix lacryma-jobi* L. [Family: Poaceae]) (Kong et al., 2021; Zhu et al., 2021), which have the effects of invigorating spleen and excreting water (“*JianPi-LiShui*), and removing toxins and dissipating mass (“*JieDu-SanJie”*). Clinical evidence from randomized clinical trials and systematic reviews showed that when used as adjunctive therapy for cancers, KLT plus chemotherapy can improve survival time, disease response rates, quality of life, and immune functions, and also reduce adverse reactions caused by chemotherapy drugs (Huang et al., 2020; Zhu et al., 2021). KLT has been recognized by other countries such as the United States and Russia, and it is the first Chinese medicinal preparation to receive approval for cancer treatment in the United States (Kong et al., 2021).

A systematic review with or without meta-analysis is often considered the highest level of evidence in the evidence-based field of healthcare (Brunström et al., 2022), and it usually serves as the cornerstone of evidence-based clinical practices (Lu et al., 2021b; Yao et al., 2021). Unfortunately, many existing systematic reviews with or without meta-analyses may be redundant, useless, confusing, or even misleading owing to overlapping or inadequate reporting, or serious methodological weaknesses (Shea et al., 2017; Chapelle et al., 2021; Hoffmann et al., 2021). Several systematic reviews with meta-analyses (Huang et al., 2020; Kong et al., 2021; Zhu et al., 2021) of KLT for cancers or conditions related to cancers have been published in recent years. For example, Huang and colleagues (Huang et al., 2020) have summarized the efficacy and safety data reported in 27 trials focusing on KLT plus platinum-based chemotherapy for advanced non-small-cell lung cancer. However, to the best of our knowledge, there are no studies that have summarized the results of these quantitative systematic reviews and evaluated their methodology simultaneously, in order to offer a collective assessment of the field’s evidence.

An overview of systematic reviews with or without meta-analyses is a method of evidence synthesis methods, and it differs from systematic reviews that intend to include primary research such as randomized controlled trials and cohort studies (Gates et al., 2020; Bougioukas et al., 2021). An overview is usually structured in a way to include systematic reviews or meta-analyses on the same health topic, to synthesize evidence from these structured reviews and to provide a more comprehensive evidence landscape (Bougioukas et al., 2021; Lunny et al., 2021). This approach is now popular with evidence-based healthcare practitioners and health policy makers, as shown by the fact that this type of evidence has been rapidly increasing in the past years (Bougioukas et al., 2021). Moreover, evidence mapping is a novel method to present evidence directly through visualization and, in turn, has been widely used in overviews (Lu et al., 2021a; Lu et al., 2021b). For instance, in the latest study (Lu et al., 2021a) by our team, an overview with evidence map was conducted to summarize the evidence from meta-analyses of Chinese medicines to treat gastric cancer. Given the aforementioned research gap, this overview was designed to map the clinical evidence on KLT for cancers or conditions related to cancers that have been reported in published meta-analyses. Meanwhile, “A Measurement Tool to Assess Systematic Reviews (AMSTAR) 2 (Shea et al., 2017; De Santis et al., 2021), a widely used tool for assessing the methodological quality of systematic reviews and meta-analyses, was employed to evaluate eligible studies in this overview.

## Methods

The present study is an overview of published systematic reviews with meta-analyses focusing on the use of KLT in treating cancers or complications of cancers. This study was completed by referring to our previous publication (Lu et al., 2021a) and relevant methodological paper (Pollock et al., 2017), and presented based on the Preferred Reporting Items for Systematic Reviews and Meta-Analyses (PRISMA) 2020 guidelines (Page et al., 2021) (Supplementary Table S1).

### Literature Search

On 25 November 2021, two bibliographic databases, PubMed and Embase, were fully searched to identify systematic reviews that were related to KLT in cancer treatment or that of conditions related to cancer. The search timespan was set from inception to the day of the search. Medical Subject Headings (MeSH) terms combined with keywords were used to establish the search strategy, without any restrictions, such as language or status of the publication. The key search terms included “Kanglaite,” “Kang-lai-te,” “Coix seed oil,” “YiYiRen,” “Yi-Yi-Ren,” “Systematic Review,” “Systematic Reviews as topic,” “Meta-analysis,” and “Meta-analysis as topic.” In addition to the database search, the reference lists of the included meta-analyses were also checked for potentially eligible studies. The complete search strategy is presented in Supplementary Table S2.

### Study Selection

Two investigators independently screened the records by performing a database search using Endnote X9 (Version X9, Clarivate Analytics). Any conflict was resolved through discussion or by consulting a third reviewer. The present overview included the studies that met each of the following criteria: 1) Participants: patients with cancer or related conditions (e.g., malignant pleural effusion) were confirmed based on cytology or pathology, regardless of other features, such as age, gender, tumor stage, nationality, or race; 2) Intervention/comparison: the control group included studies that had a common therapeutic regimen, such as chemotherapy or radiochemotherapy, whereas the trial group had a regimen of KLT injection or capsule plus the intervention of the control group; 3) Study design: published quantitative systematic reviews (i.e., pairwise meta-analyses) focusing on KLT used to treat cancers or related conditions. The concept of a meta-analysis used here is identical to that in our previous publication (Lu et al., 2021b); 4) Clinical outcomes: any synthesized efficacy (e.g., objective response rate, quality of life) or safety (e.g., gastrointestinal reactions, liver injury) outcomes reported in eligible systematic reviews were considered, regardless of the specific criteria for assessing the clinical effects; and 5) Language: only peer-reviewed meta-analyses published in English were considered. Publications or documents that did not meet the stated requirements, such as abstracts presented at meetings, network meta-analyses, qualitative systematic reviews, methodological studies, or protocol of a meta-analysis, were excluded.

### Data Extraction

Two reviewers performed the data abstraction independently, and any discrepancy was addressed by discussion. Before the formal extraction, three eligible meta-analyses were used to prepare a pilot abstraction to ensure the accuracy of the extracted data. A predesigned Microsoft Excel 2016 sheet was used to extract the following information: title, name of the first author, year of publication, country of the corresponding author, name of the journal and its impact factor (IF) in 2020, registration and protocol information, number and design of the included studies, type of cancers or conditions, number of patients enrolled, bibliographic databases that were searched (names were standardized using the common terms), details of the intervention/comparison, criteria for quality or risk of bias assessment, information on funding and conflicts of interest, synthesized clinical outcomes and corresponding effect sizes (e.g., hazard ratio (HR), risk ratio (RR), odds ratio (OR), risk difference (RD), and mean difference [MD]), as well as statistical models and I^2^ values, if reported by the original meta-analyses.

### Methodological Quality Assessment

Two independent investigators used the AMSTAR 2 tool to assess the methodological quality of KLT meta-analyses included in the present overview, and any disagreement was resolved through discussion or by consulting the third author. AMSTAR 2, consisting of 16 items, was originally developed to evaluate the methodological quality of systematic reviews/meta-analyses of randomized or non-randomized interventional studies. Although the critical domains can be adjusted, items 2, 4, 7, 9, 11, 13, and 15 were also considered as the critical domains in this overview, exactly as recommended by AMSTAR 2 developers in the original paper (Shea et al., 2017). In the overview of quantitative systematic reviews, “yes” or “no” responses were possible for items 1, 3, 5, 6, 10, 11, 12, 13, 14, 15, and 16, whereas items 2, 4, 7, 8, and 9 could be answered with a “yes,” “partial yes,” or “no.” Eventually, based on the number of responses of critical and non-critical domains, the overall methodological quality of a meta-analysis can be graded as “high,” “moderate,” “low,” or “critically low.” In the overview, similar to the previous publications (Lu et al., 2021a; Lu et al., 2021b), the percentage of “yes” <60% for an item indicated that the methodology required by that item had to be improved particularly.

### Data Analysis

Principal information of the included meta-analyses and clinical evidence derived from these studies were synthesized narratively. To evaluate the methodological quality, we calculated the frequency and percentage with the corresponding 95% confidence interval (CI) of each response for each item according to AMSTAR 2, and a radar plot was used to directly indicate the quality of each item. In addition, an evidence map was used to visualize the multi-dimensional information of each publication; the *x*-axis represented the overall methodological quality, and the *y*-axis indicated the year of publication. The size of the bubble was proportional to the total number of patients, and colored bubbles were used to label the type of cancers or related conditions. Excel 2016 (Microsoft Corporation, WA, United States) and Stata 17/SE (StataCorp, College Station, TX, United States) were used for statistical analysis. Two-sided *p* < 0.05 was considered statistically significant.

## Results

### Results of the Study Search and Screening

**FIGURE 1:**
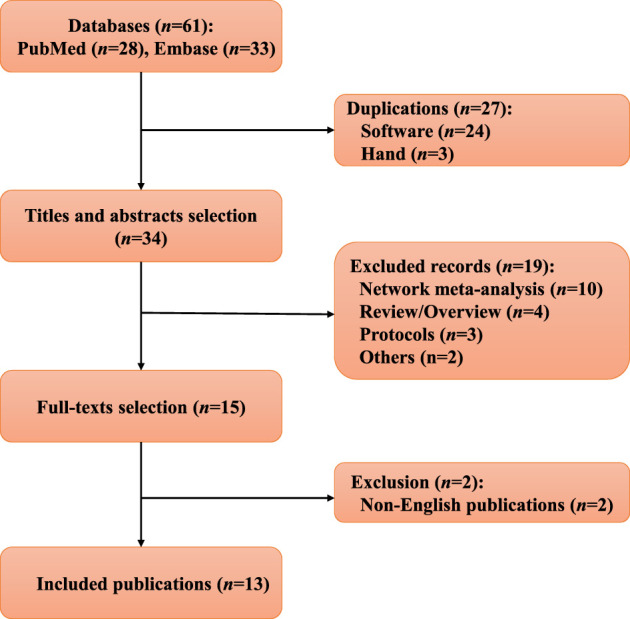
Flow chart of the study selection in this overview.

Based on bibliographic database searches, 61 records were identified from PubMed and Embase. After removing 27 duplications, 34 publications were further screened based on titles and abstracts. After retrieving full-text articles, 13 meta-analyses (Liu et al., 2008; Fu et al., 2014; Liu et al., 2014; Chen et al., 2016; Liu et al., 2019a; Liu et al., 2019b; He et al., 2020; Huang et al., 2020; Li et al., 2020; Song et al., 2020; Wen et al., 2020; Kong et al., 2021; Zhu et al., 2021) were eventually included, as no additional eligible systematic review could be supplemented from the reference lists of the included publications. A flow chart of the selection process is presented in Figure 1.

### Basic Characteristics of Included Meta-Analyses

The 13 meta-analyses that were included focused on the efficacy and safety of KLT in conditions including non-small cell lung cancer (*n* = 7, 53.85%), malignant pleural effusion (*n* = 2, 15.38%), and digestive system malignancies including hepatocellular carcinoma (n = 2), pancreatic cancer (*n* = 1, 7.69%), and digestive tract malignancy (*n* = 1). KLT combined with chemotherapy was the most common intervention in trial groups, and chemotherapy alone was the most used intervention in control groups. These systematic reviews were all conducted by authors with affiliations in China, and the years of publication ranged from 2008 to 2021. For single meta-analyses, the number of randomized controlled trials or (and) non-randomized studies ranged from 7 to 34, with an average of 21, and the total number of participants with cancer ranged from 488 to 2,964, with an average of 1,596, respectively. In terms of journals that published meta-analyses on KLT, *Frontiers in Pharmacology* had the highest IF (IF_2020_ = 5.810) and *Phytomedicine* ranked second (IF_2020_ = 5.340); the former had published two papers while the later published one. The *Journal of Cancer Research and Therapeutics* published the largest number of studies (*n* = 3, 23.08%; IF_2020_ = 1.805).

In the meta-analyses in question, the average number of databases searched was seven, PubMed/Medline and Embase were the most used English databases, whereas the China National Knowledge Infrastructure (CNKI) database was the most used Chinese database. Among them, only three meta-analyses registered their protocols on the PROSPERO website, a recognized website for registering systematic reviews and overviews (Pieper and Rombey, 2022). The Cochrane risk of bias tool (*n* = 13, 100%) was the most commonly used tool for the quality assessment of primary studies included in these systematic reviews. Regarding funding, eight (61.54%) meta-analyses received funding support, four (30.77%) stated that they did not receive any support, and one study did not report relevant information. Regarding conflicts of interest, ten (76.92%) systematic reviews had no competing interests to declare, whereas conflicts of interest were not declared in the remaining three. Details of the basic characteristics of these meta-analyses are reported in Table 1.

**TABLE 1:** Basic characteristics of included meta-analyses.

Study	Country	Journal	IF2020	Registration Information	Protocol	Number of Studies	Number of Patients	Database (Number)	Patient	Intervention	Tool for Quality Assessment	Funding	COI
Zhu et al. (2021)	China	Frontiers in pharmacology	5.810	Not mentioned	Not mentioned	20	1,293	PubMed/Medline,Embase,Cochrane Library,CNKI,CBM,WanFang,VIP (7)	Malignant pleural effusion(Lung cancer, breast cancer, etc.)	KLT + chemotherapy vs. Chemotherapy(Cisplatin 40–60mg/m2 1 week, Carboplatin 200–400 mg 1 week, etc.)	Cochrane RoB tool, Jadad	Yes	No COI exist
Kong et al. (2021)	China	Frontiers in pharmacology	5.810	Not registered	Not mentioned	12	1,046	PubMed/Medline,Embase,Cochrane Library,Web of Science,CNKI,WanFang,VIP (7)	NSCLC(III-IV)	KLT(100–200 ml/d)+EGFR-TKI vs. EGFR-TKI (Gefitinib 250 mg/d, Icotinib 375 mg/d, etc.)	Cochrane RoB tool	Yes	No COI exist
Wen et al. (2020)	China	Evidence-based complementary and alternative medicine	2.629	PROSPERO, CRD42018087094	Yes	25	2,151	PubMed/Medline,Embase,Cochrane Library,Web of Science,CNKI,CBM,WanFang,VIP (8)	Advanced NSCLC	KLT(100–200 ml/d)+chemotherapy vs. Chemotherapy(platinum + gemcitabine/docetaxel, etc.)	Cochrane RoB tool	Yes	No COI exist
Song et al. (2020)	China(Macau)	Medicine	1.889	PROSPERO, CRD42019130508	Yes	20	1,339	PubMed/Medline,Embase,Cochrane Library,Web of Science,CNKI,CBM,WanFang,VIP,Airiti Library (9)	Digestive tract malignancies(Esophageal, gastric, and colorectal cancer, III–IV) without surgery	KLT(100, 200 ml/d)+fluorouracil-based chemotherapy vs. Fluorouracil-based chemotherapy(cisplatin plus 5-fluorouracil, etc.)	Cochrane RoB tool	Yes	No COI exist
Li et al. (2020)	China	Annals of palliative medicine	2.595	PROSPERO, CRD42019142414	Yes	32	2,577	PubMed/Medline,Embase,Cochrane Library,Web of Science,CNKI,CBM,WanFang,VIP (8)	NSCLC(III-IV)	KLT(100, 200 ml)+platinum-based chemotherapy vs. Platinum-based chemotherapy(cisplatin or paraplatin and gemcitabine, etc.)	Cochrane RoB tool	Yes	No COI exist
Huang et al. (2020)	China(Macau)	Phytomedicine	5.340	Not mentioned	Not mentioned	27	2,243	PubMed/Medline,Embase,Cochrane Library,CNKI,CBM,WanFang,VIP,CSCD,Airiti Library (9)	NSCLC(III-IV)	KLT(100, 200 ml)+platinum-based chemotherapy vs. Platinum-based chemotherapy (Gemcitabine plus cisplatin, etc.)	Cochrane RoB tool, Jadad	Yes	No COI exist
He et al. (2020)	China	Journal of cancer research and therapeutics	1.805	Not mentioned	Not mentioned	7	554	PubMed/Medline,Embase,Cochrane Library,CNKI,CBM,WanFang (6)	NSCLC(III-IV)	KLT(100, 200 ml)+gefitinib vs. Gefitinib (250 mg/d)	Cochrane RoB tool	Yes	No COI exist
Liu et al. (2019a)	China	Bioscience reports	3.840	Not mentioned	Not mentioned	31	2,315	PubMed/Medline,Embase,Cochrane Library,Web of Science,CNKI,CBM,WanFang,VIP (8)	Advanced hepatocellular carcinoma	KLT(10–20 g/d)+conventional treatment vs. Conventional treatment(Oxaliplatin, etc.)	Cochrane RoB tool, MINORS	Yes	No COI exist
Liu et al. (2019b)	China	Medicine	1.889	Not mentioned	Not mentioned	16	960	PubMed/Medline,Embase,Cochrane Library,Web of Science,CNKI,CBM,WanFang,VIP (8)	Advanced pancreatic cancer	KLT(100, 200, 300–500 ml)+radiochemotherapy vs. Radiochemotherapy (Gemcitabine, γ-SBRT, etc.)	Cochrane RoB tool	No	No COI exist
Chen et al. (2016)	China	International journal of clinical and experimental medicine	—	Not mentioned	Not mentioned	10	488	PubMed/Medline,Embase,Cochrane Library,PsycINFO,CNKI,CBM,VIP (7)	Malignant pleural effusion	KLT + cisplatin vs. Cisplatin	Cochrane RoB tool	Not mentioned	No COI exist
Liu et al. (2014)	China	Journal of cancer research and therapeutics	1.805	Not mentioned	Not mentioned	34	2,964	PubMed/Medline,Embase,CNKI,WanFang (4)	Advanced NSCLC	KLT + chemotherapy vs. Chemotherapy(Gemcitabine and cisplatin, etc.)	Cochrane RoB tool	No	Not declared
Fu et al. (2014)	China	Journal of cancer research and therapeutics	1.805	Not mentioned	Not mentioned	9	608	PubMed/Medline,Embase,CNKI,WanFang (4)	Unresectable hepatocellular carcinoma	KLT(100, 200 ml)+hepatic arterial intervention vs. Hepatic arterial intervention	Cochrane RoB tool	No	Not declared
Liu et al. (2008)	China	Current therapeutic research	—	Not mentioned	Not mentioned	26	2,209	PubMed/Medline,Embase,Cochrane Library,CNKI,CBM (5)	Primary NSCLC	KLT + chemotherapy vs. Chemotherapy(Vinorelbine + cisplatin, etc.)	Cochrane RoB tool	No	Not declared

Note: CBM, Chinese biomedical literature database; CNKI, China national knowledge infrastructure database; COI, conflicts of interest; IF, impact factor; KLT, Kanglaite; MINORS, methodological index for nonrandomized studies; NSCLC, non-small cell lung cancer; PROSPERO, international prospective register of systematic reviews; RoB, risk of bias; VIP, China science and technology journal database.

### Methodological Quality of Included Meta-Analyses

**FIGURE 2:**
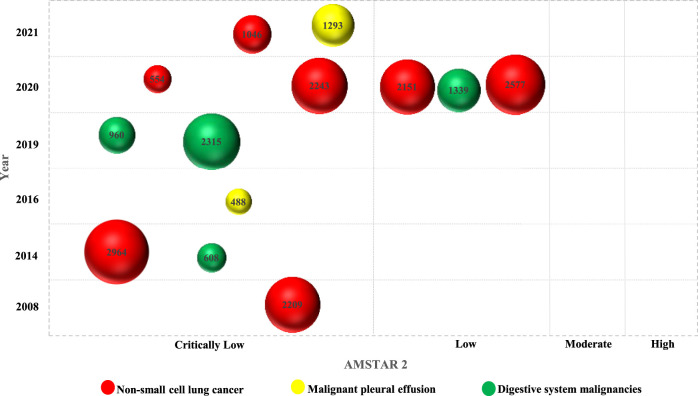
Evidence map of the methodological quality.

**FIGURE 3:**
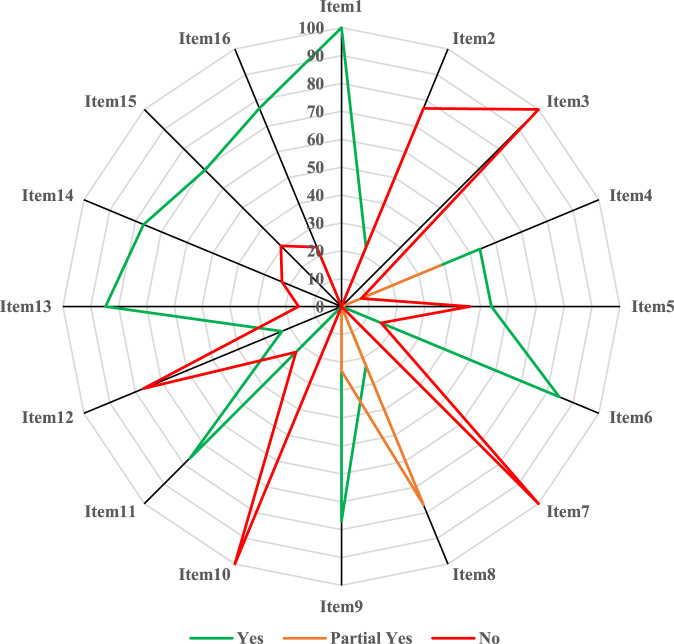
Methodological quality evaluated by the AMSTAR 2.

In terms of overall methodological quality, the quality of only three studies was low, whereas that of the other 10 meta-analyses was critically low (Figures 2, 3). Specifically, the percentages of “yes’’ response of items 2 (“*Did the report of the review contain an explicit statement that the review methods were established prior to the conduct of the review, and did the report justify any significant deviations from the protocol*’’; *n* = 3, 23.08%, 95% CI (8.18%, 50.26%)), 3 (*“Did the review authors explain their selection of the study designs for inclusion in the review*’’; *n* = 0), 4 (*“Did the review authors use a comprehensive literature search strategy*’’; n = 7, 53.85%, 95% CI (29.14%, 76.79%)), 5 (“Did the review authors perform study selection in duplicate’’; n = 7), 7 (“*Did the review authors provide a list of excluded studies and justify the exclusions*’’; n = 0), 8 (*“Did the review authors describe the included studies in adequate detail”*; n = 3), 10 (“*Did the review authors report the sources of funding for the studies included in the review*’’; n = 0), 12 (*“If meta-analysis was performed, did the review authors assess the potential impact of risk of bias in individual studies on the results of the meta-analysis or other evidence synthesis*’’; n = 0) were all less than 60% (Supplementary Table S3), which was predefined and used in our previous publications (Lu et al., 2021a; Lu et al., 2021b); they represented the major methodological weaknesses of KLT meta-analyses included in the overview.

### Kanglaite in the Management of Non-Small Cell Lung Cancer

Seven (Liu et al., 2008; Liu et al., 2014; He et al., 2020; Huang et al., 2020; Li et al., 2020; Wen et al., 2020; Kong et al., 2021) of the included systematic reviews (Supplementary Table S4) summarized the clinical effects of KLT as adjunctive therapy for the treatment of non-small cell lung cancer. The objective response rate and quality of life (i.e., performance status based on Karnofsky score) were the most reported clinical outcomes. Among them, two reviews (He et al., 2020; Kong et al., 2021) reported that KLT combined with an epidermal growth factor receptor-tyrosine kinase inhibitor (e.g., gefitinib, erlotinib) versus an epidermal growth factor receptor-tyrosine kinase inhibitor alone. For example, in a recent one by Kong et al. (2021), the authors found that KLT as an adjunctive treatment significantly improved the quality of life [OR, 2.76, 95% CI (1.73, 4.39), I^2^ = 0%; 5 trials, 365 patients], objective response rate [OR, 2.59, 95% CI (1.87, 3.58), I^2^ = 0%; 9 trials, 750 patients] as well as disease control rate [OR, 3.26, 95% CI (2.22, 4.77), I^2^ = 0%; 9 trials, 750 patients], and immunologic indicators [CD4^+^: MD, 5.36, 95% CI (3.60, 7.13), I^2^ = 91%; CD4^+^/CD8^+^ ratio: (MD, 0.18, 95% CI (0.08, 0.27), I^2^ = 65%; 6 trials, 570 patients]. Moreover, KLT reduced adverse drug reactions including nausea and vomiting (OR, 0.34, 95% CI (0.19, 0.60), I^2^ = 44%; 4 trials, 347 patients) but did not significantly influence the occurrence rate of rash [OR, 0.71, 95% CI (0.47, 1.09), I^2^ = 0%; 7 trials, 563 patients], diarrhea, and liver injury.

The other five reviews (Liu et al., 2008; Liu et al., 2014; Huang et al., 2020; Li et al., 2020; Wen et al., 2020) focused on KLT plus chemotherapy versus chemotherapy alone, and they consistently demonstrated that KLT plus chemotherapy could not only improve the objective response rate and quality of life, but could also reduce the untoward effects (e.g., nausea and vomiting, leukopenia) of chemotherapy drugs. In a review conducted by Huang *et al.* (Huang et al., 2020) and published in 2020 in *Phytomedicine*, the authors included 27 randomized trials and reported that compared with platinum-based chemotherapy, KLT combined with chemotherapy could prolong the 1-year survival rate [RR, 1.20, 95% CI (1.02, 1.43), I^2^ = 29%; 4 trials, 361 patients], enhance the objective response rate [RR, 1.45, 95% CI (1.31, 1.60), I^2^ = 0%; 27 studies, 2,243 patients], disease control rate [RR, 1.20, 95% CI (1.15, 1.26), I^2^ = 0%; 25 studies, 2066 patients], quality of life [RR, 1.32, 95% CI (1.25, 1.40), I^2^ = 0%; 21 trials, 1766 patients], immunologic indicators (i.e., CD4^+^: MD, 4.86, 95% CI (4.00, 5.73), I^2^ = 0%; CD4^+^/CD8^+^ ratio: (MD, 0.19, 95% CI (0.07, 0.31), I^2^ = 0%), and reduce toxicity including nausea and vomiting [RR, 0.46, 95% CI (0.31, 0.68), I^2^ = 0%; 11 trials, 848 patients] and leukopenia [RR, 0.36, 95% CI (0.27, 0.50), I^2^ = 0%; 13 trials, 974 patients] caused by chemotherapy drugs, but the impact on other adverse reactions such as decrease in hemoglobin [RR, 0.32, 95% CI (0.07, 1.54), I^2^ = 0%; 2 studies, 126 patients], thrombocytopenia, neurotoxicity, and liver injury was not statistically significant. The detailed pooled outcomes (including effect sizes with 95% CIs) reported in included meta-analyses can be found in Supplementary Table S4.

### Kanglaite in the Management of Malignant Pleural Effusion

Two meta-analyses (Chen et al., 2016; Zhu et al., 2021) that were included reported the effectiveness of KLT as an adjunctive treatment for malignant pleural effusion caused by cancers, such as those of the lungs and breast. A systematic review with 20 trials was published in *Frontiers in Pharmacology* in 2021 by Zhu et al. (2021), in which the authors concluded that compared with chemotherapy monotherapy, KLT plus chemotherapy could improve the objective response rate and quality of life of patients with malignant pleural effusion and reduce gastrointestinal reactions [RR, 0.79, 95% CI (0.66, 0.96), I^2^= 0%; 12 studies, 818 patients] and renal damage [RR, 0.47, 95% CI (0.23, 0.95), I^2^= 0%; 4 studies, 335 patients] caused by chemotherapy. However, the impact on chest pain [RR, 0.91, 95% CI (0.67, 1.23), I^2^= 22.8%; 7 studies, 511 patients] and myelosuppression [RR, 0.88, 95% CI (0.66, 1.17), I^2^ = 0%; 10 studies, 753 patients] was not significantly different between the two groups. The other one (Chen et al., 2016) published in 2016, it was reported that compared with cisplatin alone, KLT combined with cisplatin could promote the response rate [OR, 4.33, 95% CI (2.78, 6.75), I^2^ = 0%; 10 studies, 488 patients] and quality of life [OR, 3.07, 95% CI (1.30, 7.23), I^2^ = 0%; 3 studies, 114 patients] of patients with malignant pleural effusion, and reduce the incidence of nausea and vomiting [OR, 0.22, 95% CI (0.10, 0.48), I^2^ = 0%; four studies, 150 patients]. However, chest pain relief [OR, 0.51, 95% CI (0.22, 1.18), I^2^ = 79%; three studies, 102 patients] and fever [OR, 1.29, 95% CI (0.51, 3.29), I^2^ = 0%; three studies, 130 patients] were not significantly different between KLT plus cisplatin versus cisplatin monotherapy.

### Kanglaite in the Management of Digestive System Malignancies

A total of four meta-analyses (Fu et al., 2014; Liu et al., 2019a; Liu et al., 2019b; Song et al., 2020) provided clinical evidence of KLT as an adjunctive treatment in the treatment of digestive system malignancies. Of them, two reviews (Fu et al., 2014; Liu et al., 2019a) focused on hepatocellular carcinoma. In a recent meta-analysis published in 2019 by Liu et al. 2019a), the reviewers included 31 studies with 2,315 patients with advanced hepatocellular carcinoma. They found that compared with conventional treatment (e.g., transcatheter arterial chemoembolization, transhepatic arterial embolization) alone, KLT plus conventional treatment could improve overall survival [e.g., 6-month overall survival: OR, 2.85, 95% CI (1.42, 5.71), I^2^ = 0%; six trials, 366 patients], overall response rate [OR, 2.57, 95% CI (2.10, 3.16), I^2^ = 0%; 27 studies, 2035 patients], disease control rate [OR, 3.10, 95% CI (2.42, 3.97), I^2^= 0%; 25 studies, 1,927 patients], quality of life [OR, 3.80, 95% CI (3.01, 4.80), I^2^ = 0%; 19 studies, 1,449 patients], immune indicators (e.g., CD3^+^, CD4^+^), clinical symptoms (e.g., appetite, hepatalgia). Moreover, this combination could reduce several adverse reactions including hepatotoxicity [OR, 0.40, 95% CI (0.25, 0.66), I^2^ = 0%; 7 trials, 418 patients], fever, leukopenia, nausea and vomiting, and thrombocytopenia. Another study (Fu et al., 2014) published in 2014 in the *Journal of Cancer Research and Therapeutics* reported that compared with hepatic arterial intervention alone, KLT plus hepatic arterial intervention could promote the objective response rate [OR, 1.80, 95% CI (1.18, 2.75), I^2^ = 0%; 7 trials, 502 patients] and quality of life [OR, 3.22, 95% CI (1.36, 7.60), I^2^= 0%, 3 trials, 96 patients], and relieve the pain [OR, 2.57, 95% CI (1.65, 3.99), I^2^= 0%, five trials, 383 patients] of patients with unresectable hepatocellular carcinoma.

Liu et al. (2019b) published a meta-analysis in 2019, focusing on KLT plus radiochemotherapy compared with radiochemotherapy alone for advanced pancreatic cancer. They reported that KLT as an adjunctive treatment could improve the 1-year overall survival [OR, 2.58, 95% CI (1.12, 5.93), I^2^ = 23%, 3 trials, 144 patients], disease response rate [e.g., objective response rate: OR, 2.16, 95% CI (1.58, 2.94), I^2^ = 0%, 15 trials, 897 patients], quality of life [OR, 3.68, 95% CI (2.36, 5.75), I^2^ = 0%; six trials, 368 patients], pain relief rate [OR, 3.70, 95% CI (2.23, 6.14), I^2^ = 0%; 6 trials, 281 patients], and weight gain rate [OR, 3.69, 95% CI (2.22, 6.13), I^2^ = 0%; 6 trials, 288 patients]; decrease tumor markers including carbohydrate antigen-199 (MD, -4.49, 95% CI (-6.57, -2.40), I^2^ = 0%; two trials, 94 patients) and carcinoembryonic antigen in patients; and reduce adverse effects such as gastrointestinal reactions (OR, 0.68, 95% CI (0.47, 0.98), I^2^ = 0%; nine trials, 594 patients), leukopenia, thrombocytopenia, myelosuppression, and nephrotoxicity. However, there was no significant difference in outcomes between KLT plus radiochemotherapy versus radiochemotherapy alone with respect to adverse effects such as diarrhea (OR, 0.67, 95% CI (0.33, 1.34), I^2^ = 21%; 3 studies, 154 patients), neurotoxicity (OR, 0.80, 95% CI (0.42, 1.51), I^2^ = 0%; five studies, 316 patients), anemia, rash, and fatigue.

The meta-analysis published in 2020 by Song et al. (2020) summarized the clinical evidence on KLT combined with fluorouracil-based chemotherapy versus fluorouracil-based chemotherapy. The synthesized outcomes revealed that KLT plus chemotherapy could improve the objective response rate [OR, 1.35, 95% CI (1.18, 1.54), I^2^ = 0%; 17 trials, 1,227 patients], disease control rate [OR, 1.18, 95% CI (1.11, 1.25), I^2^ = 0%; 17 trials, 1,227 patients], quality of life [OR, 1.73, 95% CI (1.50, 2.00), I^2^ = 20%; 11 studies, 815 patients], and immunologic function [e.g., CD3^+^: MD, 7.67, 95% CI (5.71, 9.63), I^2^ = 24%; CD4^+^: MD, 5.51, 95% CI (1.99, 9.02), I^2^ = 68%; 3 trials, 173 patients] of patients with advanced digestive tract malignancies including esophageal, gastric, and colorectal cancer, and reduce the rate of occurrence of anemia [OR, 0.41, 95% CI (0.23, 0.75), I^2^ = 0%; 4 trials, 231 patients], nausea and vomiting [OR, 0.41, 95% CI (0.28, 0.61), I^2^ = 0%; 9 studies, 596 patients], diarrhea, myelosuppression, leukopenia, neutropenia, thrombocytopenia, hepatotoxicity, and neurotoxicity.

## Discussion

Chinese medicines as adjunctive treatment can improve the effectiveness and reduce the toxicity of chemotherapeutic drugs (Wang et al., 2020a; Lu et al., 2021a); thus, the products of Chinese medicine are valuable in treating patients with cancers and improving their quality of life. According to the global cancer statistics in the year 2020, lung cancer caused 1.8 million (18%) deaths and is the leading cause of all deaths worldwide, and liver cancer ranked third with 8.3% of deaths (Sung et al., 2021). KLT is a Chinese medicine preparation from Coix seeds that has been approved to treat lung and liver cancer in China (Zhu et al., 2021). In this overview with evidence map, 13 systematic reviews with meta-analyses of KLT published from 2004 to 2021 were included and evaluated. Among these 13 studies, eight and two among them focused on the clinical effects of KLT in the treatment of non-small cell lung cancer and hepatocellular carcinoma, respectively. The remaining studies were for the treatment of other cancers or related complications, such as malignant pleural effusion.

The findings of the included meta-analyses suggested that compared with conventional treatment alone, KLT combined with conventional treatment (e.g., chemotherapy drugs) could promote various outcomes in patients with cancer (e.g., lung cancer, hepatocellular carcinoma, and pancreatic cancer) or malignant pleural effusion. Specifically, on the one hand, KLT adjunctive therapy could improve clinical indices such as the overall survival, disease response rate (e.g., objective response rate, disease control rate), and quality of life; on the other hand, KLT could improve the values of laboratory biomarkers, such as immune indicators including CD4^+^, CD4^+^/CD8^+^ ratio. Moreover, KLT could decrease the incidence rate of various adverse reactions, such as gastrointestinal reactions (e.g., nausea and vomiting), leukopenia, anemia, thrombocytopenia, and hepatotoxicity, which are common when using conventional chemotherapy drugs. Meanwhile, preclinical studies support these clinical outcomes, as several studies have revealed that KLT has several modes of exerting antitumor effects, such as promoting cancer cell apoptosis, inhibiting migration and proliferation, affecting mitosis, reversing multidrug resistance, and improving cellular immunity (Huang et al., 2014; Yang et al., 2018). Also, the clinical outcomes resulting from KLT intervention could be explained on the basis of the traditional Chinese medicine theory of reinforcing health and eliminating pathogens (“FuZheng-QuXie”) (Liu et al., 2020; Lu et al., 2021a). However, most of the meta-analyses included in this overview were of critically low quality based on the AMSTAR 2 tool, and only three studies were evaluated as low.

Considering items 3, 7, and 10 with the worst performance (percentages of “yes” equal to “0”) as examples, the importance of the methodology required by AMSTAR 2 was illustrated below. Item 3 requires reviewers to consider the impact of study design for inclusion and to provide reasonable reasons, because the inclusion of only one study design (e.g., randomized controlled trials) may lead to incomplete and inaccurate effect estimates when non-randomized interventional studies (e.g., cohort studies) are also available (Shea et al., 2017), particularly for the evidence synthesis of long-term safety outcomes, as these data are mainly derived from real-world studies (Lu et al., 2021a). Item 7 expects reviewers to provide a list of excluded original studies and to justify their exclusion during full-text selection (Shea et al., 2017). The rationale of this requirement is to promote the transparency of literature selection and ensure that end users can judge the potential influence after excluding certain publications (Lu et al., 2021a). Systematic reviews/meta-analyses often aim to synthesize results from randomized trials, while the concept of “garbage in, garbage out” applies to the inclusion of biased studies in a meta-analysis (Jüni et al., 1999), and questionable funding support (e.g., unreasonable commercial funding) can influence or even distort the outcomes of clinical trials (Lundh et al., 2018); therefore, item 10 requires reviewers to report the sources of funding of the primary studies included in the systematic reviews in question in order to help judge the reliability of the pooled outcomes. The major methodological flaws involving the abovementioned items 3, 7, 10 as well as items 2, 4, 5, 8, and 12 of AMSTAR 2 tool, should be addressed and significantly improved in future meta-analyses assessing KLT as adjunctive treatment in cancer and related conditions. In addition, some items, such as items 9 (“satisfactory evaluation of the risk of bias”), 11 (“appropriate method for statistical combination”), 14 (“detailed explanation and discussion of heterogeneity”), 15 (“adequate investigation and discussion of small study bias”), and 16 (“sources of conflict of interest in review”) also need to be considered with more attention, because the percentage of “yes*”* among these items was lower than 80% even though higher than the predefined threshold (60%). However, although AMSTAR 2 now is widely accepted and used to assess the methodological quality of systematic reviews/meta-analyses (De Santis et al., 2021; Pieper et al., 2021), studies have shown that AMSTAR 2 tends to give a low or critically low quality to systematic reviews of various interventions across different clinical specialties (De Santis et al., 2021), therefore, the discrimination ability of AMSTAR 2 for the meta-analyses of different quality may need to be improved in addition to enhancing the methodological quality of relevant articles.

Our research has several strengths. To the best of our knowledge, this study is the first overview focusing on KLT for the treatment of cancers and associated conditions, in which the methodological quality of the included meta-analyses was evaluated using AMSTAR 2 and the evidence mapping method was used to present the overall methodological quality. Second, an overview consists of several key steps, including literature selection, data abstraction, and quality assessment. All of these steps were performed by at least two independent reviewers, thereby ensuring the accuracy of data to support our results (Carroll et al., 2013; Wang et al., 2020b). Third, the clinical evidence summarized in this overview can be used as a source for clinical decision making, and the identified knowledge gaps can be used to better design future studies of KLT, including systematic reviews and clinical trials. Several meta-analyses (Huang et al., 2020; Li et al., 2020; Wen et al., 2020) have stated that the reporting of methodological details (e.g., randomization or blinding methods, allocation concealment) of the included clinical trials was inadequate and unclear, and almost all trials were conducted in China and published in Chinese, which limited the generalizability of the results from the meta-analyses. Moreover, long-term survival data are lacking in the relevant trials (Huang et al., 2020; Li et al., 2020; Kong et al., 2021). Therefore, future trials should be carefully designed and conducted rigorously, and they should be reported per the requirements of the corresponding reporting guidelines, such as Consolidated Standards of Reporting Trials (CONSORT) 2010 (Schulz et al., 2010) and its extensions (Cheng et al., 2017; Juszczak et al., 2019). As recommended previously (Lu et al., 2021b), meta-analyses of KLT should be conducted and reported using the AMSTAR 2 tool and PRISMA 2020 guidelines, respectively. As health technology assessment and economic evaluation are being gradually incorporated for informed clinical decision making and drug pricing (Huang et al., 2022), and as value-based healthcare concept is emerging (Reitblat et al., 2021), future studies should not only report survival data from long-term follow-up, but they should also pay more attention to the cost-effectiveness of KLT as an adjunctive treatment, as of the whole field of traditional medicine, as well (Chen, 2022).

This overview also has some limitations. First, it was not registered prospectively on PROSPERO, due to the delay expected because of the steep increase in the number of studies pertaining to COVID-19. However, the prospective protocol may be not particularly useful, as our research team is very familiar with the overview using the evidence mapping approach (Lu et al., 2021a; Lu et al., 2021b; Lu et al., 2022). Second, similar to other published overviews (Lu et al., 2021a; Michels et al., 2022), only two large bibliographic databases, namely, PubMed and Embase were searched rather than searching other databases, such as the Cochrane Library, which is collected in PubMed, although may have a time lag. Third, only quantitative systematic reviews published in English were included and Chinese papers were excluded. This exclusion may seem inappropriate given the language background of members of the research team; however, considering almost all trials are from China and published in Chinese (Huang et al., 2020; Li et al., 2020), the pooled clinical evidence summarized in this overview may not significantly change even if KLT meta-analyses published in Chinese are included. Moreover, the status quo of the methodological quality of KLT meta-analyses identified in this overview may be over-optimistic, because a recent meta-research (Cao et al., 2021) has proved that the quality of meta-analyses published in Chinese was relatively worse than those published in English. However, in order to perfectly address this question, a subsequent meta-epidemiological study (Sterne et al., 2002) comparing KLT meta-analyses published in Chinese and English may be a good option.

## Conclusion

This study is the first overview with evidence map that provides a comprehensive evidence landscape based on published meta-analyses focusing on KLT for the treatment of cancers and related conditions. Although existing evidence shows that KLT as an adjunctive treatment is effective and safe to treat non-small cell lung cancer, malignant pleural effusion, and digestive system malignancies (e.g., hepatocellular carcinoma), the methodological quality of the meta-analyses of KLT included in this overview was poor. Well-designed and fully reported randomized trials and meta-analyses of KLT use in cancer and cancer-related conditions should be conducted in the future in order to confirm and/or expand the results presented in this overview.

## Data Availability

The original contributions presented in the study are included in the article/Supplementary Material.
