# Supplementary material for: Kanglaite (Coix Seed Extract) as Adjunctive Therapy in Cancer: Evidence Mapping Overview Based on Systematic Reviews With Meta-Analyses
Source: Front Pharmacol. 2022 Aug 12;13:901875. doi: 10.3389/fphar.2022.901875 (PMC9413959; doi:10.3389/fphar.2022.901875)
Supplement: Supplementary file 3 [file Table2.docx]

**Supplementary S2.** Search strategy

**(1) PubMed**

#1 OR/

"Kang-lai-te"[Supplementary Concept]

"Kanglaite"[Title/Abstract]

"Kang-lai-te"[Title/Abstract]

"Coix seed oil"[Title/Abstract]

"YiYiRen"[Title/Abstract]

"Yi-Yi-Ren"[Title/Abstract]

#2 OR/

"Systematic Review"[Publication Type]

"Systematic Reviews as topic"[Mesh]

"Meta-analysis"[Publication Type]

"Meta-analysis as topic"[Mesh]

"Systematic review"[Title/Abstract]

"Meta-analysis"[Title/Abstract]

#3 #1 AND #2

**(2) Embase**

#1 OR/

'kang lai te'/exp

'coix seed oil'/exp

'yi yi ren'/exp

'kanglaite':ab,ti

'kang-lai-te':ab,ti

'coix seed oil':ab,ti

'yiyiren':ab,ti

'yi-yi-ren':ab,ti

#2 OR/

'systematic review'/exp

'meta analysis'/exp

'systematic review':ab,ti

'meta-analysis':ab,ti

#3 #1 AND #2
